# Novel anti-biofouling bioactive calcium silicate-based cement containing 2-methacryloyloxyethyl phosphorylcholine

**DOI:** 10.1371/journal.pone.0211007

**Published:** 2019-01-17

**Authors:** Jae-Sung Kwon, Myung-Jin Lee, Ji-Young Kim, Dohyun Kim, Jeong-Hyun Ryu, Sungil Jang, Kwang-Mahn Kim, Chung-Ju Hwang, Sung-Hwan Choi

**Affiliations:** 1 Department and Research Institute of Dental Biomaterials and Bioengineering, Yonsei University College of Dentistry, Seoul, Republic of Korea; 2 Department of Orthodontics, Institute of Craniofacial Deformity, Yonsei University College of Dentistry, Seoul, Republic of Korea; 3 Department of Conservative Dentistry, Yonsei University College of Dentistry, Seoul, Republic of Korea; 4 BK21 PLUS Project, Yonsei University College of Dentistry, Seoul, Republic of Korea; 5 Department of Oral Biology, Yonsei University College of Dentistry, Seoul, Republic of Korea; Yonsei University, REPUBLIC OF KOREA

## Abstract

Calcium silicate-based cements (CSCs) are commonly used for endodontic procedures; however, their antibacterial effects are limited. The objective of this study was to develop a 2-methacryloyloxyethyl phosphorylcholine (MPC)-incorporated CSC with improved antibacterial properties, while maintaining the original advantageous features of CSC. MPC was incorporated into a commercial CSC (Endocem MTA) at 0 wt% (control), 1.5%, 3.0 wt%, 5.0 wt%, 7.5 wt%, and 10 wt%. The setting time, compressive strength, water sorption, and glycerol contact angle were measured. Protein absorption was measured and bacterial adhesion on the surface was evaluated using *Enterococcus faecalis*. The bactericidal effect was examined by the disc diffusion test. Mineralization ability was assessed based on calcium ion deposition, as assessed by alizarin red staining, after immersion into Hank’s balanced salt solution for 7 days. High concentrations of MPC in CSC (7.5 wt% and 10 wt%) increased the setting time, reduced compressive strength, and reduced wettability. MPC (3 wt%) had greater protein repellent and anti-biofouling effects than those of control and test materials (*P* < 0.001). However, no bactericidal effect was observed for any control or test materials. There was greater calcium ion deposition on the surface of MPC-supplemented CSC than on the control (*P* < 0.001). The addition of 3 wt% MPC polymer to CSC confers protein-repellent properties and reduced bacterial attachment, with the potential for improved mineralization.

## Introduction

Mineral trioxide aggregate (MTA), a calcium silicate-based cement (CSC), was initially developed as an endodontic material for perforation repair and root-end filling [1,2]. Since then, various types of CSCs have been introduced and used in a number of endodontic procedures, such as regenerative endodontics, partial pulpotomy, apexogenesis, apexification, perforation repair, endodontic surgery, and root canal filling [3,4]. CSCs have even been formulated into restorative materials for dentin replacement or repair [5]. This wide range of applications is possible owing to the favorable properties of CSCs, which include a strong sealing ability, biocompatibility, and the potential to induce hard tissue formation [1,6]. Additionally, recent studies have demonstrated the bioactivity of CSCs, which induce mineralization via the formation of apatite precipitates [7].

An ideal endodontic material should have adequate physical, biological, and antimicrobial properties. Despite the increasing use of CSCs, bacterial infection following treatment remains a significant complication [8,9]. Moreover, some studies have reported that CSCs could cause problems related to inflammatory reactions in the pulp or other related tissues [10]. Hence, it is necessary to develop CSCs with improved antibacterial properties for the prevention of bacterial ingress following the application of the material.

Zwitterionic materials are a biologically inspired family of materials characterized by high dipole moments and highly charged groups, as they possess both cationic and anionic groups, while the overall charge of the material remains neutral [11]. 2-Methacryloyloxyethyl phosphorylcholine (MPC) is a commonly used zwitterionic material with a methacrylate and phospholipid polar group side chains, providing a highly hydrophilic surface that can resist protein absorption and bacterial adhesion [12]. MPC has been applied in various biomedical applications [12,13], including recent applications in dental materials, such as dental composite materials [14] and dentin bonding agents [15].

The aim of this study was to develop an MPC-supplemented CSC with improved antibacterial properties via anti-biofouling effects. We hypothesized that the combination of MPC and CSC would achieve a superior antibacterial effect, while maintaining other characteristics, such as biocompatibility and mineralization potential.

## Materials and methods

### Preparation of MPC-incorporated CSC

Commercially available MPC powder (Sigma-Aldrich, St. Louis, MO, USA) and CSC (Endocem MTA, Maruchi, Wonju-si, Korea) were used in this study. MPC powder was mixed into CSC at various weight percentages (1.5, 3.0, 5.0, 7.5, and 10.0 wt%), and CSC without MPC was used as a control. The compositions of the experimental and control materials are summarized in Table 1. All samples were mixed in accordance with the manufacturer’s instructions for CSC.

**Table 1:** Composition of materials in the control and experimental groups.

	Calcium silicate-based cement (Endocem MTA), wt%	2-Methacryloyloxyethyl Phosphorylcholine (MPC), wt%
**Control**	100	0
**1.5% MPC**	98.5	1.5
**3% MPC**	97.0	3.0
**5% MPC**	95.0	5.0
**7.5% MPC**	92.5	7.5
**10% MPC**	90.0	10.0

### Setting time

The setting time was determined according to the international standard ISO 6876 [16] and a previous study [17]. Each material was mixed and placed in a circular stainless steel mold of 10 mm in diameter and 2 mm in height. The assembly was placed in a cabinet at 37°C and 95% relative humidity. At 180 s after the end of mixing, a Gilmore-type indenter of 430 g in weight with a flat-end needle of 1.0 mm in diameter was carefully lowered vertically onto the surface of the material and was allowed to remain there for 5 s. To determine the approximate setting time, the indentations were repeated at 30-s intervals until the needle failed to make a complete circular indentation on the material. This process was then repeated, starting the indentation at 30-s before the approximate setting time that was initially measured, making indentations at 10-s intervals to determine the setting time of the material.

### Compressive strength

As there is no established standard method for determining the compressive strength of CSCs for endodontic materials, a test was adapted from the method described in the international standard ISO 9917–1 [18]. Each material was mixed according to method described above and placed in a split stainless steel mold of 6 mm in diameter and 4 mm in height. Following the setting time, samples were removed from the molds and checked visually for any air voids or chipped edges, where such defective samples were discarded. The compressive strength was tested using a universal testing machine (Model 5942; Norwood, Instron, MA, USA) at a crosshead speed of 1.0 mm/min. When both planes were in contact with the samples, the compressive load was recorded until a loading failure point was reached. The maximum load at loading failure was used to calculate the compressive strength of the CSC samples using the equation in ISO 9917–1 (Eq (1)):

$$\sigma =\frac{4P}{\pi d^{2}}$$ (1)

where, σ is the compressive strength, *P* is the maximum applied load in Newtons, and *d* is the mean diameter of the cylindrical samples in millimeters.

### Water sorption

The water sorption test was adapted from a previous study [17]. Each material was placed in a mold of 20 mm in diameter and 1.5 mm in height. The mean diameters of the samples were calculated by measuring two diameters, and the mean thicknesses of samples were calculated by measuring three equally spaced points on the circumference. These values were then used to calculate the volume (V) of all samples (in 0.01 mm^3^). Then, samples were weighted using an analytical balance (accurate to 0.01 mg) (XS105; Mettler-Toledo AG, Greifensee, Switzerland) with a reproducibility of 0.1 mg until a constant mass (m_1_) was obtained. All the samples were then immersed in distilled water and placed in a water bath maintained at 37°C for 24 h. Samples were then blotted until free from visible moisture, waved in the air for 10 s, and weighed to determine the final mass (m_2_). Water sorption (*W*_sp_) values were calculated using the following equation (Eq (2)) in units of g/mm^3^:

$$W_{sp}=\frac{m_{2}-m_{1}}{V}$$ (2)

### Wettability

Wettability was measured in accordance with the methods of previous studies [19,20]. Glycerol solution was chosen as the reference liquid, since it is an ideal blood analog with a similar viscosity to the normal range of human blood [19,21]. Each material was placed in a mold with a diameter of 15 mm and a thickness of 1 mm to form disc-shaped samples. The static contact angle 10 s after the addition of 2 μL of glycerol (Sigma-Aldrich) on the sample surface was then measured using a video contact angle goniometer (SmartDrop; Femtobiomed Inc., Gyeonggi-do, Korea).

### Protein adsorption

Protein adsorption was tested according to a previously established method [22]. Each material was placed in a mold with diameter of 15 mm and a thickness of 2 mm to form disc-shaped samples. All samples were immersed into fresh phosphate-buffered saline (PBS; Gibco, Grand Island, NY, USA) for 1 h at room temperature and immediately immersed into a protein solution of either bovine serum albumin (BSA; Pierce Biotechnology, Rockford, IL, USA) or brain heart infusion (BHI; Difco, Sparks, MD, USA) broth (at a concentration of 2 mg of protein per mL of PBS and a volume of 100 μL). After incubation at 37°C for 1 h, the samples were gently rinsed twice with fresh PBS. After 4 h of incubation under sterile humid conditions at 37°, any protein that was not adhered was removed by washing twice with PBS. The amount of protein adhered to samples was measured using 200 μL of micro-bicinchoninic acid (Micro BCA^TM^ Protein Assay Kit; Pierce Biotechnology) followed by incubation at 37°C for 30 min. Proteins adsorbed on the surfaces were quantitatively analyzed following the measurement of the absorbance at 562 nm using a micro-plate reader (Epoch, BioTek Instruments, Winooski, VT, USA).

### Bacterial attachment, colony forming units, and viability

Bacterial analyses were performed using *Enterococcus faecalis* (ATCC 29212) cultured in BHI (Difco) in an incubator at 37°C.

Each material was placed in a mold with a diameter of 10 mm and a thickness of 2 mm to form disc-shaped samples and 1 mL of bacterial suspension (1 × 10^8^ cells/mL) was placed on each disc in a 24-well plate. They were then incubated at 37°C for 24 h.

For the microscopic examination of attached bacteria, bacteria on the samples were fixed with 2% glutaraldehyde-paraformaldehyde in 0.1 M PBS for at least 30 min at room temperature. The samples were post-fixed with 1% OsO_4_ dissolved in 0.1 M PBS for 2 h, dehydrated in an ascending gradual series of ethanol, treated with isoamyl acetate, and subjected to critical point drying (LEICA EM CPD300; Leica, Wien, Austria). Then, the discs were coated with Pt (5 nm) using an ion coater (ACE600; Leica) and images were obtained by scanning electron microscopy (FE-SEM; Merin, Carl Zeiss, Oberkochen, Germany) at 2 kV.

To evaluate bacterial colony forming units (CFU), adherent bacteria were harvested in 1 mL of BHI by sonication (SH-2100; Saehan Ultrasonic, Seoul, Korea) for 5 min. Of this bacterial suspension, 100 μL was spread onto a BHI agar plate and incubated at 37°C for 24 h. Then, the total number of colonies was counted.

Finally, the viability of adherent bacteria was examined by staining using a Live/dead Bacterial Viability Kit (Molecular Probes, Eugene, OR, USA), according to the manufacturer’s protocols. Equal volumes of Syto 9 dye and propidium iodide, which stain live and dead bacteria, respectively, from the kit were mixed thoroughly. Of the mixture, 3 μL was added to 1 mL of the bacterial suspension prepared as described above. After 15 min of incubation at room temperature in the dark, the stained samples were observed under a confocal laser microscope (LSM700; Carl Zeiss, Thornwood, NY, USA). Live bacteria appeared green and dead bacteria appeared red.

### Disc-diffusion test

The disc diffusion test, adapted from a previous study [23], was used to measure bactericidal properties. The same bacterial culture of *Enterococcus faecalis* (ATCC 29212) was used for this study. First, 100 μL of bacterial culture suspension (1 × 10^4^ cells/mL) was spread uniformly on BHI agar plates. Each material was placed in a mold with a diameter of 10 mm and a thickness of 2 mm to form disc-shaped samples. Solid samples of test materials were directly placed in contact with the surface of the agar. A filter-paper disc with same diameter as the disc-shaped sample was placed on the surface of the agar plate soaked with 20 μL of 5.25% sodium hypochlorite (NaOCl) as a positive control. The choice of the positive control was based on a previous study that used NaOCl as an effective irrigation solution to effectively dissolve organic matter and kill microbes at concentrations of higher than 1% to 2% [23]. The plates were incubated for 24 h at 37°C and the inhibition zones around each sample were measured with Vernier calipers (Mitutoyo, Kawasaki, Japan).

### Mineralization ability

The disc-shaped control and 3% MPC CSC samples with diameters of 10 mm and a thickness of 2 mm were immersed in Hank’s balanced salt solution (HBSS; Welgene, Gyeongsangbuk-do, South Korea) for 1 day and 7 days. Samples were then dried in a 100°C oven overnight (12 h) and coated with carbon. Images were obtained by FE-SEM (Merin, Carl Zeiss, Oberkochen, Germany) at 2 kV.

Additionally, samples immersed in HBSS were rinsed with calcium-free, Dulbecco’s phosphate-buffered saline (DPBS; Gibco), followed by exposure to 40 mM alizarin red stain solution at a pH of 4.2 (adjusted by 10% ammonium hydroxide) for 10 min under gentle agitation. After staining, samples were rinsed with DPBS for 1 h to remove non-specific staining and 10% cetylpyridinium chloride assay was applied. The quantitative calcium deposition on each sample was calculated following the measurement of absorbance at 562 nm using a micro-plate reader (Epoch, BioTek Instruments).

### Statistical analysis

For all statistical analyses, IBM SPSS version 23.0 (IBM Korea Inc., Seoul, Korea) for Windows was used, with data from at least three independent experiments. The results obtained for the control and experimental groups were analyzed by one-way analysis of variance (ANOVA) followed by Tukey’s test. Comparisons between two groups were performed using the independent *t-*test. *P* < 0.05 was considered statistically significant.

## Results

### Physical and chemical properties

**Fig 1:**
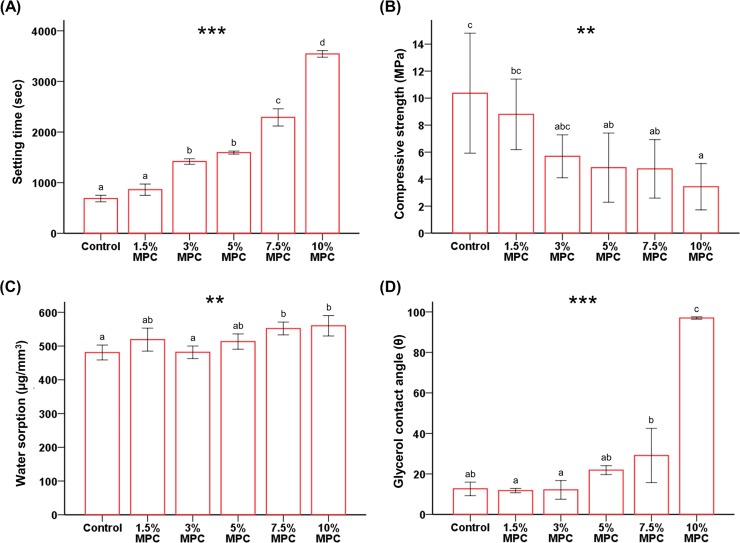
Comparison of physical and chemical properties between groups. (A) Setting time. (B) Compressive strength. (C) Water sorption. (D) Glycerol contact angle. Different letters above bars indicate significant differences. **P < 0.01, ***P < 0.001 for comparisons between calcium silicate-based cements (CSC) with different concentrations of 2-methacryloyloxyethyl phosphorylcholine (MPC).

Generally, setting time increased as the amount of MPC added to CSC increased. The setting time for 10% MPC (3543.33 ± 65.06 s) was nearly five times that of the control (686.66 ± 65.06 s) (*P* < 0.001, Fig 1). However, there were no significant differences between the control and 1.5% MPC samples. Additionally, despite a significantly longer setting time for 3% MPC than for 1.5% MPC, there were no significant differences between 3% MPC and 5% MPC samples.

Compressive strength generally decreased as the amount of MPC added to CSC increased and was over two-fold lower for 10% MPC samples (3.43 ± 1.71 MPa) than the control (10.36 ± 4.44 MPa) (*P* < 0.01, Fig 1). However, there were no significant differences between the control, 1.5% MPC, and 3% MPC samples.

In terms of water sorption, there were no significant differences between the control, 1.5% MPC, 3% MPC, and 5% MPC samples (Fig 1). Both 7.5% MPC and 10% MPC samples resulted in higher values for water sorption than those of the control (*P* < 0.01).

Finally, the wettability of the samples was measured by the contact angle using glycerol (Fig 1). It was evident that the average contact angle of the control sample was about 12 degrees. There were no significant differences between the control, 1.5% MPC, 3% MPC, and 5% MPC samples. The 10% MPC sample resulted in a dramatic increase in contact angle to nearly 100 degrees (97.00 ± 0.55 degrees) (*P* < 0.001).

Overall, it was evident that the 7.5% MPC and 10% MPC samples resulted in significant declines in physical and chemical properties compared to those of the control; therefore, these concentrations were not used in further experiments.

### Protein adsorption

**Fig 2:**
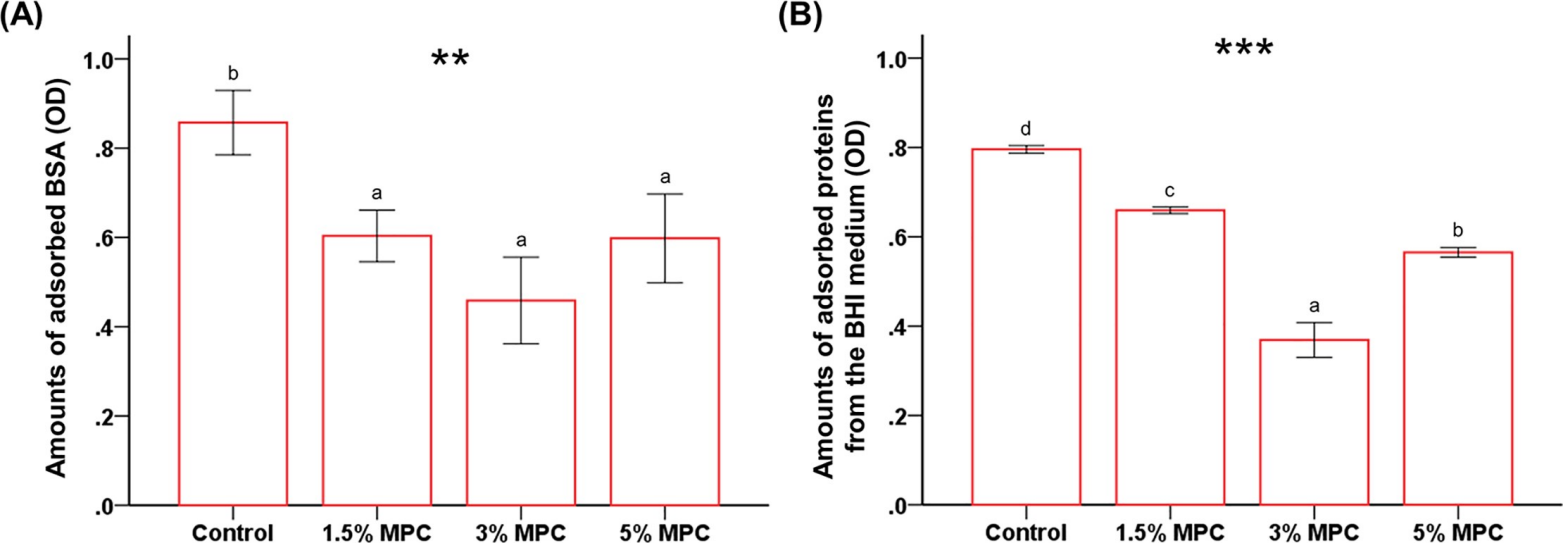
Comparison of the optical density (OD) of protein adsorption between groups. (A) Adsorbed bovine serum albumin (BSA). (B) Non-specific protein adsorbed from brain heart infusion (BHI) medium. Different letters above bars indicate significant differences. **P < 0.01, ***P < 0.001 for comparisons between calcium silicate-based cements (CSC) with different concentrations of 2-methacryloyloxyethyl phosphorylcholine (MPC).

The amount of adsorbed BSA was significantly lower on 1.5% MPC, 3% MPC, and 5% MPC than on the control (*P* < 0.01, Fig 2). In terms of test samples, BSA adsorption was the lowest for 3% MPC (optical density of 0.45 ± 0.09) followed by 1.5% MPC and 5% MPC (0.60 ± 0.05 and 0.59 ± 0.09, respectively), though the differences were not statistically significant. The amount of proteins adsorbed from BHI medium, and the results were similar to those obtained for BSA adsorption. Again, 3% MPC showed significantly lower adsorption compare to those of the other samples, followed by 5% MPC, 1.5% MPC, and the control, in order (*P* < 0.001, Fig 2).

### Bacterial attachment, colony forming units, and viability

**Fig 3:**
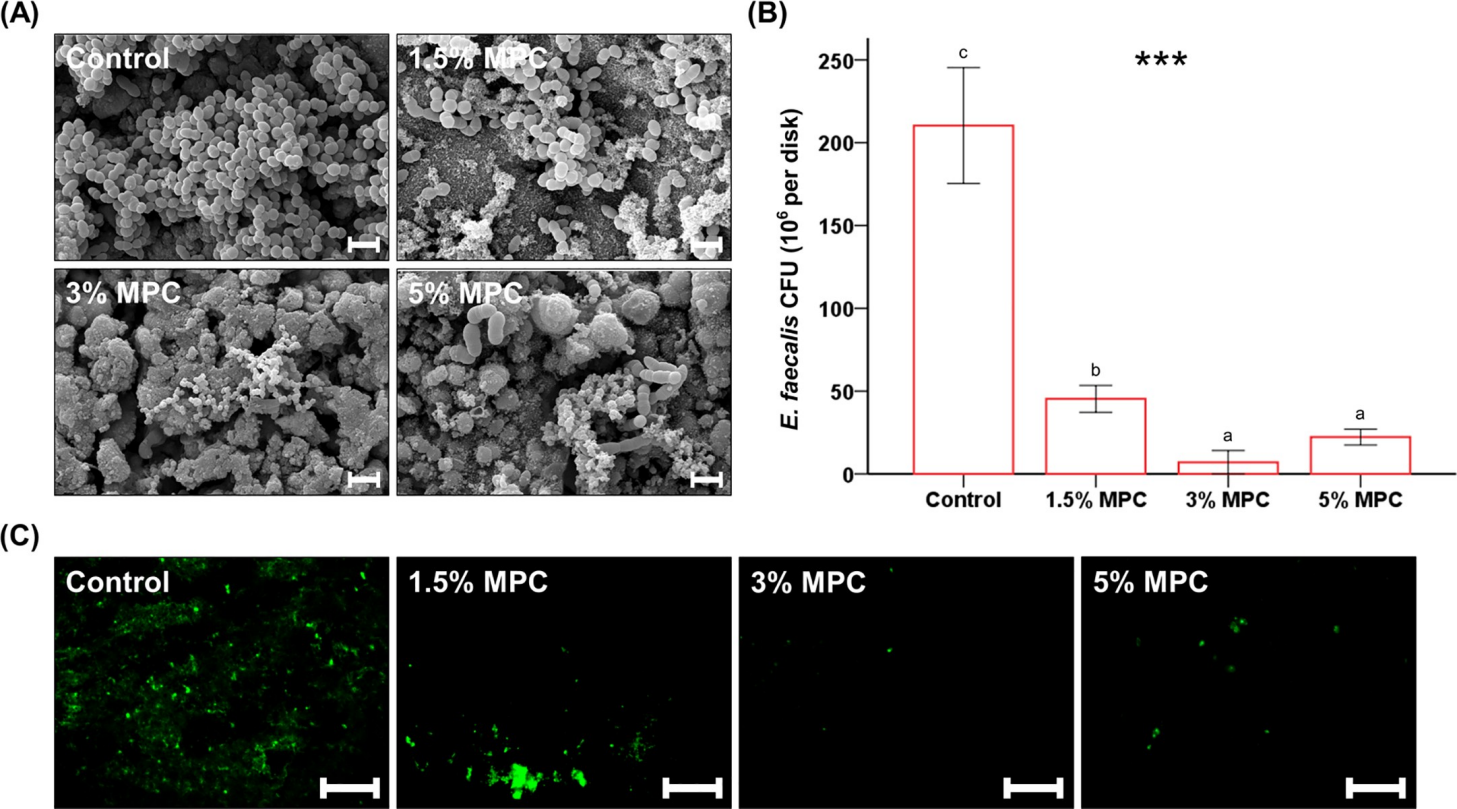
Comparison of bacterial attachment, colony forming units, and viability between groups. (A) Qualitative scanning electron microscopy images of *Enterococcus faecalis* attached to the surfaces of control and experimental groups at a magnification of 5,000×. Scale bar represents 2 μm. (B) Colony-forming units (CFUs) for *E*. *faecalis* attached on the surfaces of calcium silicate-based cement (CSC) with different concentrations of 2-methacryloyloxyethyl phosphorylcholine (MPC). Different letters above bars indicate significant differences. ***P < 0.001 for comparisons between CSC with different concentrations of MPC. (C) Representative live/dead staining images of bacteria attached to the surfaces of control and experimental groups. Scale bar represents 500 μm.

FE-SEM images showed bacteria with typical appearances of *Enterococcus faecalis* cocci located on granular structures of CSCs for the control, 1.5% MPC, 3% MPC, and 5% MPC samples (Fig 3). Fewer bacteria were detected on 1.5% MPC, 3% MPC, and 5% MPC samples than on the control.

The result was confirmed by a quantitative analysis of CFU counts. The CFU count decreased significantly by the addition of MPC to CSC; the values were significantly lower for 1.5% MPC, 3% MPC, and 5% MPC samples than for the control (*P* < 0.001, Fig 3). Both 3% MPC and 5% MPC showed significantly lower CFUs compare to that of the 1.5% MPC sample.

Viability staining results confirmed the above findings. Fewer live bacteria (visible as green staining) were attached to samples containing MPC, particularly for 3% MPC and 5% MPC compared to 1.5% MPC (Fig 3). However, there was no evidence for dead bacteria (visible as red staining) on any sample.

### Bactericidal properties

**Fig 4:**
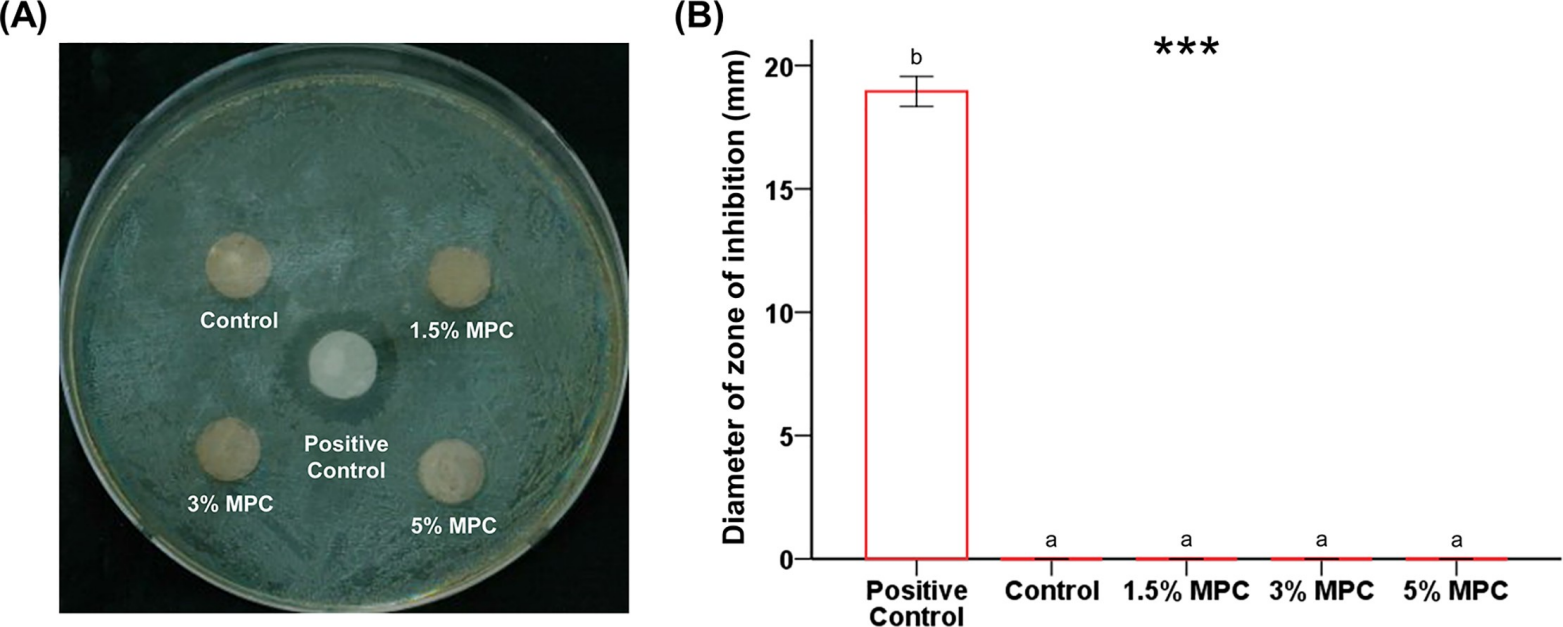
Comparison of bactericidal properties between groups. (A) Disc diffusion test using calcium silicate-based cement (CSC) with different concentrations of 2-methacryloyloxyethyl phosphorylcholine (MPC) on agar plates with evenly spread *Enterococcus faecalis* for 24 h. (B) The diameters of the zone of inhibition on the agar plate were measured. Different letters above bars indicate significant differences. ***P < 0.001 for comparisons between CSC with different concentrations of MPC.

Bactericidal properties of the samples were examined using a disc diffusion test method where samples were placed on agar plate with an evenly spread culture of *E*. *faecalis* (Fig 4). Large zones formed for the positive control of NaOCl (diameter of 18.95 ± 0.60 mm). However, no zones formed for any of the samples (*P* < 0.001).

### Mineralization ability

**Fig 5:**
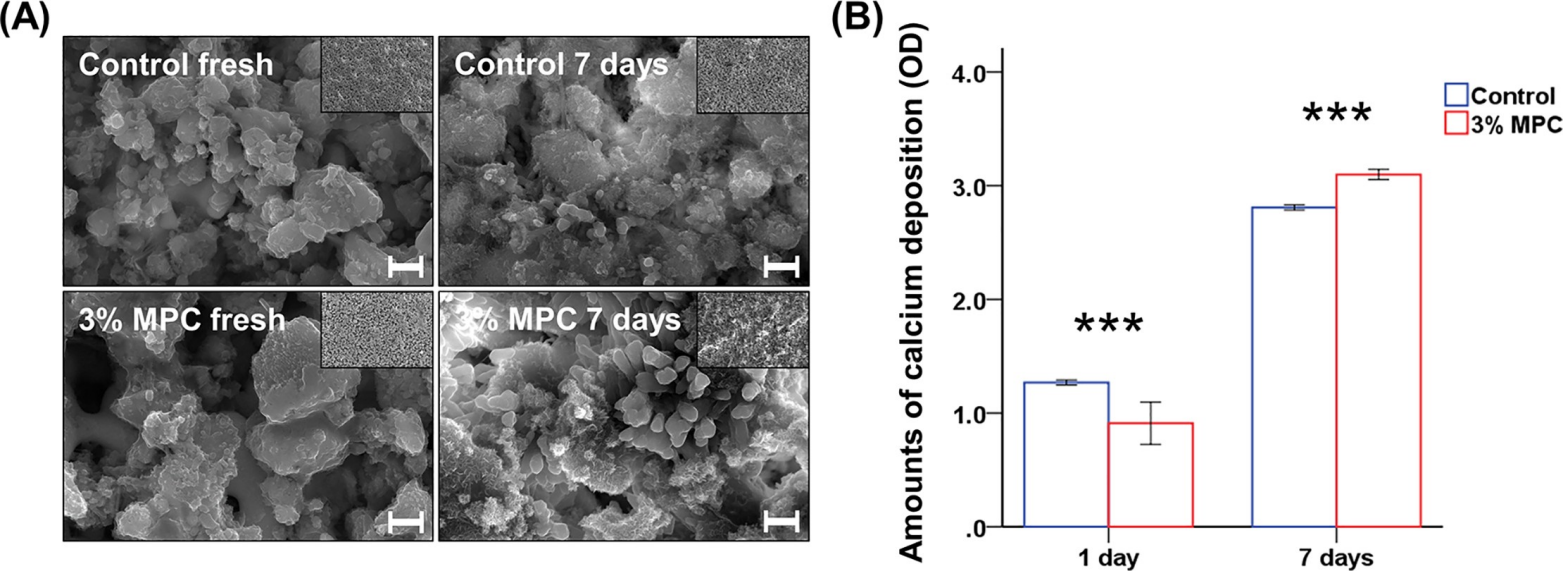
Comparison of mineralization ability between groups. (A) FE-SEM images of the control and calcium silicate-based cement (CSC) with 3% 2-methacryloyloxyethyl phosphorylcholine (MPC), before and 7 days after immersion into the HBSS solution at a magnification of 10,000×. Scale bar represents 10 μm. Top right corner of each image shows a magnified version (1,000×). (B) Calcium deposition on the control and CSC with 3% MPC at 1 day and 7 days after immersion into HBSS. ***P < 0.001 for comparisons between CSC without MPC and CSC with 3% MPC.

Bioactivity of the control and 3% MPC was evaluated by the ability to form mineralized structures on the surface after immersion in an HBSS solution for 7 days. The surfaces of the samples were first examined by FE-SEM (Fig 5). Before immersion into HBSS, typical granular structures of CSC were observed for both the control and 3% MPC samples. Similar results were obtained for the control sample following immersion in HBSS for 7 days, with no obvious differences in structure. However, 3% MPC samples immersed in HBSS for 7 days showed an aggregated irregular spherulite-like structure. Electron dispersive X-ray spectroscopy indicated that the structure included Ca and P; the average atomic percentages of Ca and P were 15.86% and 0.16%, respectively, for the spherulite-like structure, and 17.43% and 0.12% for the surrounding structure. Hence, the Ca/P ratio of the spherulite-like structure was approximately 99, while the Ca/P ratio of surrounding structure was approximately 145.

The mineralization of the control and 3% MPC samples was confirmed by Alizarin red staining before and after HBSS immersion for 7 days (Fig 5). At 24 h (1 day) after immersion, there was significantly greater calcium on the control than the 3% MPC sample (*P* < 0.001). However, the opposite results were obtained after 7 days of immersion; significantly greater calcium was present on the 3% MPC sample than the control sample (*P* < 0.001). The increase in calcium, as indicated by optical density, was 1.54 for the control and 2.19 for the 3% MPC sample from day 1 to day 7 of the experiment.

## Discussion

This is the first application of the zwitterionic material MPC to improve the antibacterial properties of CSC via anti-biofouling. We successfully incorporated MPC into CSC, which not only provided an added anti-biofouling property but also maintained or even improved the original advantages of CSC, such as its mineralization ability.

Commercially available CSC (Endocem MTA) was used in this study. Its biocompatibility and osteogenicity are similar to those of ProRoot MTA, with a shorter setting time and increased resistance to washout [24,25]. Indeed, the control in this study showed a short setting time of around 11 min. However, the addition of MPC into CSC caused a significant increase in setting time to nearly 1 h for 10% MPC. A long setting time is a clinical disadvantage as it would increase patient chair-time, which may not be ideal for restless or uncooperative patients (e.g., pediatric patients) and would consequently increase the risk of contamination [23,26].

Other physical properties, including the compressive strength, water sorption, and glycerol contact angle, were evaluated in accordance with relevant international standards or previous literature [17–21]. Compressive strength is an indicator of the physical strength of the material, and the contact angle of glycerol is related to surface features of the material, needed to adhere to surrounding tissues. Studies have suggested that glycerol is an ideal blood analogue, with similar viscosity, and the lower wettability (higher contact angles) against glycerol is correlated with a lower sealing ability and material penetration into dentinal tubules [19,21]. The results of this study indicated that high concentrations of MPC are not ideal in terms of maintaining the advantageous features of CSC.

Previous studies have demonstrated that the incorporation of MPC results in a hydrophilic surface, which consequently leads to greater resistance to protein absorption than that of hydrophobic surfaces [13]. In this study, we assessed absorption using two protein types, BSA and mixed proteins present in bacterial BHI culture medium. Similar results were obtained, in which all test groups exhibited significantly lower protein absorption than that of the control, while 3% MPC resulted in the lowest amount of protein absorption for both types of proteins. This result was expected based on the structure of MPC, which has a phospholipid polar group in the side chain [13]. Phospholipid molecules generally consist of hydrophilic heads that are attracted to water and hydrophobic tails that are repelled by water. Hence, MPC-incorporated CSC maintained a low contact angle with glycerol for contents up to 5 wt%. MPC phospholipids orient themselves into a bilayer when in contact with water, so that the non-polar tails face the inner area of the bilayer and the polar heads face outward to interact with water, resulting in high hydrophilicity [13]. When the MPC polymer is exposed to a protein solution, the unique structure of MPC would allow a large amount of free water to be present around the phosphorylcholine group, whereas there would be no bound water in the hydrated MPC [12]. The addition of MPC to CSC results in protein-repellent properties as the presence of bound water would promote protein adsorption, whereas the presence of free water would repel proteins [27]. However, we did not observe a clear positive correlation between the amount of MPC added to CSC and the protein repellent property, as 5% MPC showed greater protein absorption than that of 3% MPC. This result is consistent with previous studies of MPC incorporated into dental composite resin [28], polymethyl methacrylate [29], and polyethylene [30], in which protein-repellent properties are markedly decreased at high concentrations of MPC due to a disturbance in the polymerization system that results in gelation [29,30].

Bacterial infection during or after root canal treatment would result in significant complications, while a previous study has demonstrated that proteins, such as collagen-binding protein, would contribute to the adhesion of *E*. *faecalis* to endodontic tissues [31]. Hence, the protein-repellent properties of CSC would be ideal for the resistance of bacterial adhesion; indeed, we observed significantly less bacterial adhesion on the surfaces of all MPC-incorporated CSCs; CFU patterns were very similar to previously described protein absorption results. Additionally, there was no evidence for dead bacteria in a live/dead bacterial assay, indicating that bacteria were prevented from attaching to the surface, rather than being killed on the surface. This result was confirmed by a disc diffusion test that considered the bactericidal effect of the material. Despite the protein repellent property and reduced CFU counts on MPC-incorporated CSC, none of the test or control materials resulted in the formation of a zone of inhibition. The result was in agreement with those of previous studies demonstrating that CSC (identical or equivalent to the material used in this study) fails to kill or inhibit the growth of *E*. *faecalis* [26]. *E*. *faecalis* was used in this study, as it is the most clinically relevant bacteria to endodontic disease and is the most frequently isolated microorganism from infected endodontic tissues, both before and after treatment [32]. In clinical situations, the proton pump of *E*. *faecalis* would reduce the intracellular pH, and the buffering capacity of dentin would influence the surrounding pH [32]. Hence, antibacterial activity against *E*. *faecalis* cannot be explained by a high pH alone, often cited to explain the effectiveness in some of endodontic materials, including CSC; this may explain the lack of a bactericidal effect in this study. An alternative strategy may be required to prevent the growth of *E*. *faecalis*, and the addition of MPC to CSC certainly conferred an antibacterial property by preventing attachment via protein-repellent properties.

The aim of this study was not only to provide an improved antibacterial effect by preventing bacterial attachment in CSC using MPC but also to maintain the original advantageous features of CSC, including mineralization potential [33]. Hence, the presence of mineralized components was examined on the control and 3% MPC following immersion in HBSS solutions for 7 days. At 1 day, there was significantly greater calcium on the control than 3% MPC. The result may be due to the fact that CSC is a calcium hydroxide-based material; therefore, the control material with more CSC than that of the 3% MPC sample may have had a greater amount of calcium the surface [33]. However, in 7 days, the 3% MPC had higher levels of calcium on the surface than those of the control. FE-SEM imaging of 3% MPC indicated the presence of aggregated irregular spherulite-like structures. The structures were composed of Ca and P, and Ca/P ratio was significantly lower than that of the surrounding structure. Exposure of bioactive materials to physiological solutions, such as HBSS, would result in the precipitation of a ‘bone-like’ apatite layer, which is a thin layer with calcium and phosphate [34]. Previous studies have demonstrated that endodontic materials, such as CSC, would possess different levels according to the type of material and the design of the *in vitro* study [35]. The period of 1 week may not have been sufficiently long to induce mineralization on the CSC surface. However, irregular spherulite-like structures were present along with an increase in calcium deposition on 3% MPC. A low Ca/P ratio has been linked to amorphous calcium phosphate formation in relation to apatite precursors [35]. The polarity of endodontic materials would also be important for mineralization, as calcium ions along with other ions in hydroxyapatite of root canal dentin are polar in nature [21]. Additionally, it has been indicated that negatively charged polar groups on endodontic material surfaces would have a catalytic effect on the nucleation of the apatite layer or apatite precursors [34]. Hence, it is possible that the structure of MPC, with a polar phospholipid side chain orientated so that polar heads face outward and interact with liquid and the non-polar tail region faces the inner area of the bilayer, resulted in the improved formation of apatite precursors by aiding mineralization on MPC-incorporated CSC.

Owing to the nature of *in vitro* experiments, complications in the oral and endodontic environment, including salivary flow and complex interactions between materials and surrounding tissues, were not evaluated and should be considered in future *in vivo* or clinical studies. Despite this limitation, the present study clearly indicated that the addition of an appropriate amount of MPC to CSC confers protein-repellent properties and reduced bacterial attachment. The addition of 3 wt% MPC polymer was optimal in terms of both anti-biofouling properties and the maintenance of the advantageous features of CSC, with the potential for improved mineralization.

## Conclusion

The incorporation of MPC in CSC resulted in improved antibacterial properties by anti-biofouling effects, which were linked to the protein-repellent properties of MPC. The addition of the MPC polymer at 3 wt% provided anti-biofouling properties, while maintaining the physical properties of CSC and improving the mineralization potential. Hence, CSC containing MPC may provide solution to bacterial ingress during clinical application of the CSC, which would prevent inflammatory reactions in the pulp or other related tissues. Also, improved mineralization of CSC containing MPC is expected to increase the formation of apatite precipitate, and therefore results in strong sealing ability as restorative materials in clinic.

## Supporting information

S1 File All data used in this study. (PDF)
