# Supplementary material for: Novel anti-biofouling bioactive calcium silicate-based cement containing 2-methacryloyloxyethyl phosphorylcholine
Source: PLoS One. 2019 Jan 17;14(1):e0211007. doi: 10.1371/journal.pone.0211007 (PMC6336247; doi:10.1371/journal.pone.0211007)
Supplement: S1 File — (PDF) [file pone.0211007.s001.pdf]

	Setting time (sec)	Compressive strength (Mpa)	Water sorption (µg/mm3)	Contact angle (degree)	BSA (OD)	BHI (OD)	CFU	Zone of inhibition (mm)	Ca deposition (OD)	
									1 day	7 days
**Control**	620	17.52	477.59	13.3	0.94	0.8	227	0	1.2	2.8
	690	9.48	460.38	15.6	0.83	0.79	192	0	1.2	2.9
	750	5.89	504.54	9	0.81	0.8	236	0	1.3	2.8
		7.81					216	0	1.2	2.8
		11.12					221	0	1.3	2.8
							248	0	1.3	2.8
							279	0	1.3	2.8
							192	0	1.3	2.8
							169	0	1.3	2.8
							156	0	1.3	2.8
							181	0		
							207	0		
								0		
								0		
								0		
								0		
								0		
								0		
								0		
**1.5% MPC**	800	11.94	490.96	10.6	0.66	0.67	59	0		
	795	6.01	556.79	12.7	0.54	0.66	47	0		
	990	6.98	509.08	12	0.61	0.65	40	0		
		7.91					43	0		
		11.15					57	0		
							42	0		
							49	0		
							51	0		
							38	0		
							49	0		
							33	0		
							36	0		
								0		
								0		
								0		
								0		
								0		
								0		
								0		
**3% MPC**	1360	7.1	502.76	10	0.54	0.33	15	0	1	3.1
	1470	7.22	474.07	8.9	0.48	0.38	19	0	1.1	3
	1425	3.82	467.72	17.4	0.35	0.4	17	0	1.1	3.1
		4.23					15	0	1.1	3.1
		6.09					3	0	1.1	3.1
							2	0	0.7	3.1
							3	0	0.7	3.1
							3	0	0.7	3.1
							2	0	0.7	3.2
							1	0	0.7	3.2
							1	0		
							3	0		
								0		
								0		
								0		
								0		
								0		
								0		
								0		
**5% MPC**	1560	6.13	536.71	21.2	0.7	0.55	31	0		
	1600	7.39	491.34	20.1	0.58	0.57	26	0		
	1620	6.55	511.67	24.3	0.51	0.58	25	0		
		2.05					23	0		
		2.12					22	0		
							19	0		
							21	0		
							17	0		
							24	0		
							18	0		
							27	0		
							14	0		
								0		
								0		
								0		
								0		
								0		
								0		
								0		
**7.5% MPC**	2100	2.69	548.36	15						
	2340	6.95	534.97	30.7						
	2430	3.89	572.51	41.6						
		7.21								
		3.04								
**10% MPC**	3480	4.37	556.02	97.5						
	3540	5.71	592.23	96.4						
	3610	2.21	531.98	97.1						
		1.39								
		3.51								
**Positive control (NaOCl)**								19		
								18		
								18		
								19		
								20		
								19		
								19		
								20		
								20		
								18		
								19		
								19		
								19		
								19		
								19		
								19		
								19		
								18		
								19		
								19		
